# Quantitative Analysis of Transcription Termination via Position-Selective Labeling of RNA (PLOR) Method

**DOI:** 10.3390/ijms24054934

**Published:** 2023-03-03

**Authors:** Ping-Yi Chien, Lingzhi Gao, Yu Liu

**Affiliations:** State Key Laboratory of Microbial Metabolism, School of Life Science and Biotechnology, Shanghai Jiao Tong University, Shanghai 200240, China

**Keywords:** transcription termination, single-round transcription, PLOR, riboswitch RNA

## Abstract

T7 RNA polymerase is the most widely used enzyme in RNA synthesis, and it is also used for RNA labeling in position-selective labeling of RNA (PLOR). PLOR is a liquid–solid hybrid phase method that has been developed to introduce labels to specific positions of RNA. Here, we applied PLOR as a single-round transcription method to quantify the terminated and read-through products in transcription for the first time. Various factors, including pausing strategies, Mg^2+^, ligand and the NTP concentration at the transcriptional termination of adenine riboswitch RNA have been characterized. This helps to understand transcription termination, which is one of the least understood processes in transcription. Additionally, our strategy can potentially be used to study the co-transcription behavior of general RNA, especially when continuous transcription is not desired.

## 1. Introduction

RNA is transcribed by RNA polymerase (RNAP) in cells, and then involves in various cellular activities, including transferring genetic information, regulating gene expression, and catalyzing reactions as enzymes and cofactors [1,2,3,4,5,6,7]. Transcription is a highly regulated process in which RNAP initiates transcription after binding to a promoter in DNA, elongates nascent RNA and then releases RNA after reaching a terminator [8,9,10]. Transcription termination is the release of RNAP from DNA, it may occur when RNAP finishes transcribing DNA with the production of full-length RNA, or meets a termination signal with the production of abortive RNA [8,9,10]. Early termination is sometimes caused by a regulatory protein, such as Rho or by a specific folding of nascent RNA [10,11,12,13,14,15]. In the former, Rho is an ATP-dependent translocase that can translocate along nascent RNA to induce the release of RNA from the transcription system. In the latter, transcriptional termination takes place at the specific folding of nascent RNA, such as a consecutive U segment following a GC-rich hairpin [10,11,12,13,14,15]. A transcription-regulatory riboswitch RNA is usually located at the 5′ untranslated region of mRNA in bacteria, where a riboswitch serves to modulate transcription termination in response to changes in the environment by adopting alternative structures after binding with its target ligand. During transcription, the nascent riboswitch RNA folds into at least two mutually exclusive structures: an anti-terminator and a terminator. The interaction between a riboswitch and its specific ligand ignites structural switching, leading to transcriptional regulation.

Transcription termination quantification is important for us to identify transcription termination elements or factors, understand the interaction or regulation involved in transcription and compare termination efficiency among diverse polymerases or different termination strategies. In addition, quantifying transcription termination can be used to differentiate between different species and elucidate the mechanism of transcriptional control. Quantifying transcription termination of a single-round transcription provides accuracy to evaluate the performance of a transcription system. Several methods have been developed to analyze termination in single-round transcription reactions. Heparin or rifampicin has been added to the transcriptions to prevent re-initiation and achieve single-round in vitro transcription, in which ^32^P-radiolabeled NTPs are used to significantly improve the sensitivity of detecting transcription termination via polyacrylamide gel electrophoresis (PAGE) [12,13,14,15,16,17,18]. However, rifampicin or heparin is efficient to prevent re-initiation only at high concentrations, and their potential effects on transcription initiation and elongation are uncertain [19]. Additionally, ^32^P radiolabels increase the risk for experimental personnel, which is not satisfactory for conventional labs. Chung et al. developed a hybridization-based method, in which double-labeled DNA is designed to hybridize the abortive and full-length transcripts [20]. Lim et al. adopted a PEG-coated quartz surface to immobilize the elongation complex to achieve single-round transcription [21]. Passalacqua et al. delicately achieve single-pass transcription by removing the promoter sequence from DNA templates using a restriction enzyme [19].

**Figure 1:**
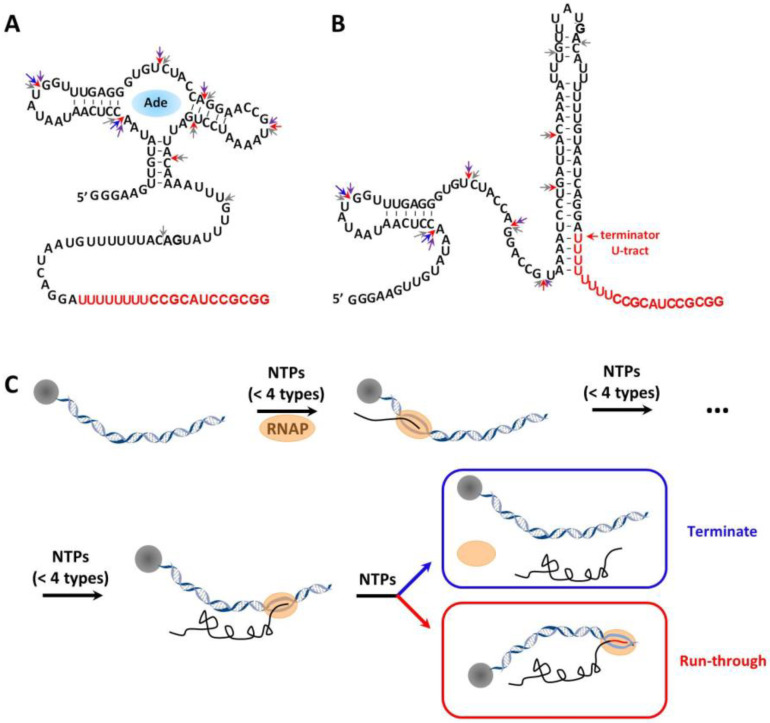
Schematic illustration of quantifying transcription termination of adenine riboswitch RNA via PLOR. (**A**,**B**) The secondary structures of adenine riboswitch RNA in the presence (**A**) and absence of adenine (**B**). Adenine is shown as a blue ball. The nucleotides in the terminator U-tract are shown in red. The pause positions at 3-step, 6-step, 8-step and 10-step PLOR reactions are marked by blue, purple, red and grey arrows, respectively. (**C**) The solid–liquid hybrid phase transcription in PLOR is divided into multiple steps by omitting certain types of NTPs. The 5′ biotin-labeled DNA templates (grey ribbons) are immobilized on streptavidin-coated agarose beads (grey beads). The RNAP and nascent RNA are shown by orange spheres and black lines, respectively. When RNAP proceeds along the terminator sequence, RNAP either dissociates from DNA and produces the terminated products (in blue box) or RNAP continues transcription to produce full-length RNA products (in red box).

Here, we extended the application of position-selective labeling of RNA (PLOR) to quantify the transcription termination of adenine riboswitch RNA (Figure 1A,B). Riboswitch RNA is located at the untranslated region of mRNA, and it consists of an aptamer and an expression platform. The aptamer domain can recognize its specific ligand and regulate gene expression by altering structures of the expression platform. In the case of *pbuE* adenine riboswitch RNA from *Bacillus subtilis*, its structures and transcription-regulatory function are dependent on the presence of adenine (Figure 1A,B). Whether transcription terminates or continues at the sites in red with the production of terminated (100 nt) or full-length RNA (120 nt) is dependent on the formation of the terminator U-tract (Figure 1C). We here tested the effects of various factors on the transcription of adenine riboswitch via PLOR. PLOR has been developed to incorporate labels into specific positions of RNA using a “pause-restart” mode [22,23,24]. The basic strategy of PLOR is to halt RNAP at chosen positions by depleting specific types of NTP(s) from the reaction mixture. PLOR is initiated by adding RNAP and less than four types of NTPs to the solid-phase DNA template, and RNAP pauses at the position where the missing NTP(s) is required. In PLOR, the biotin-tagged DNA template is immobilized on streptavidin-coated beads to enable the removal of excessive NTPs via solid–liquid phase extraction (SPE) and to allow the addition of new NTP batches in PLOR (Figure 1C). Thorough rinsing to remove the residual NTPs from the previous step is critical for eliminating cross-contamination in PLOR. After the removal of residual NTPs and the addition of new batch of NTPs, transcription in PLOR restarts and then pauses at the position that requires missing NTP again. Such “pause-restart” transcription in PLOR is template-directed and can be completed in multiple steps by omitting certain types of NTPs in individual steps [22,23,24]. This is essentially different from traditional in vitro transcription, in which four types of NTPs (ATP, CTP, GTP and UTP) incubate with RNAP and DNA template during the whole transcription to ensure highly processive RNA elongation. It is worth pointing out that compared with in vitro transcription, the ratio of DNA to NTP in the PLOR termination stage (usually ~1) is much lower (usually >10,000 in in vitro transcription), and the temperature used in PLOR is lower (25 °C for PLOR and 37 °C for in vitro transcription), which can effectively avoid re-initiation of transcription and hence generate a single transcript per DNA molecule hypothetically. Additionally, in our hands, no products generated from PLOR re-initiation were detected. In this work, PLOR was applied to analyze termination efficiency of transcription for the first time. Instead of using radiolabeled NTPs, as in most single-pass transcriptional strategies reported previously, we detected the terminated and full-length adenine riboswitch by using either unlabeled or fluorophore-labeled NTPs. Fluorophore-labeling ensures analysis of termination efficiencies with higher sensitivity. However, the usage of fluorophore NTPs increases the experimental cost and decreases the overall yields of PLOR because of the low tolerance of T7 RNAP for NTPs with bulky fluorophores. In PLOR, the DNA templates are immobilized in the solid phase, which can be recycled to measure the early termination of adenine riboswitch at various experimental conditions, including different PLOR strategies, adenine, Mg^2+^ and the NTP concentration. Our strategy can potentially be applied to other RNA and provides valuable clues to control transcription termination by adjusting environmental conditions.

## 2. Results

The adenine riboswitch RNA used in this work is from *Bacillus subtilis*, and it contains an aptamer domain and an expression platform [25]. The secondary structures of the adenine riboswitch RNA with and without binding adenine are shown in Figure 1A,B, respectively. In the absence of adenine, the riboswitch RNA adopts the structure containing a terminator U-tract, which triggers early termination and produces ~100 nt of terminated RNA (Figure 1B,C). On the contrary, in the presence of adenine, riboswitch RNA preferentially adopts the structure without forming the terminator, which allows the RNA polymerase to transcribe the whole DNA template and generates 120 nt full-length RNA (Figure 1A,C) [13,25,26]. In order to observe the early termination of the adenine riboswitch RNA, different experimental conditions, including four “pause-restart” modes of PLOR, 0~10 mM adenine, 0.5~26 mM Mg^2+^ and 0.5~10X NTPs, were tested.

### 2.1. The Effects of Pausing Modes on Transcription Termination of Adenine Riboswitch RNA

**Figure 2:**
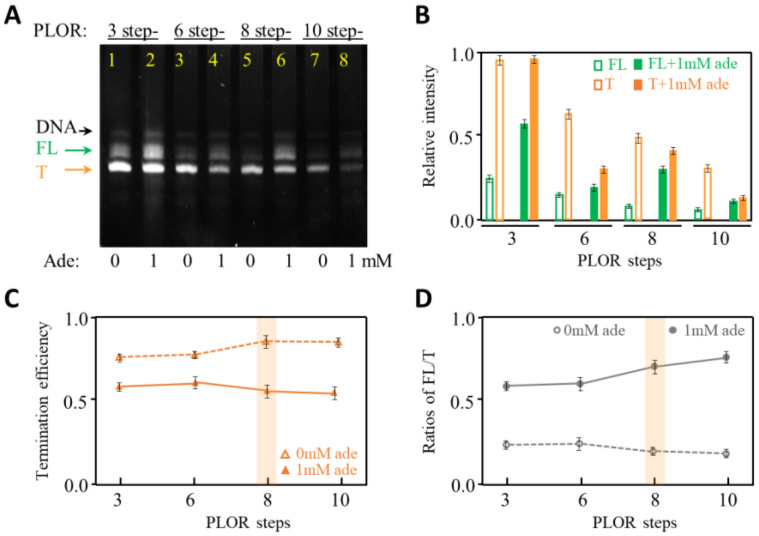
Transcription termination studies of adenine riboswitch without and with adenine in different PLOR reactions. (**A**) 12% denaturing PAGE gel image of the crude products generated from 3-step, 6-step, 8-step and 10-step PLOR reactions. Either 0 or 1 mM adenine was added in these single-round transcription reactions. The full-length and terminated RNA products are marked by FL and T, respectively. DNA dissociated from the solid-phase beads was observed in the gel. The gel was irradiated under UV 260 nm. (**B**) The relative intensities of FL (in green) and T (in orange) in the absence (in open columns) and presence of 1 mM adenine (in solid columns). The experiments were repeated three times. (**C**) The termination efficiency of adenine riboswitch in different PLOR reactions in the absence (in open triangles) and presence of 1 mM adenine (in solid triangles). (**D**) The ratios of FL to T of transcribing adenine riboswitch by different PLOR reactions in the absence (in open dots) and presence of 1 mM adenine (in solid dots).

Single-pass transcription is performed in the PLOR reactions. The enzyme used in PLOR is T7 RNAP. T7 RNAP is the most widely used enzyme in in vitro transcription and has been used to transcribe RNA that ranges from tens to thousands of nucleotides (nt) in length [24,27,28,29]. The single-subunit T7 RNAP has a distinct advantage of regulating transcriptional process by limiting NTP addition, which facilitates PLOR to flexibly pause at different positions as desired [22,23,24]. In this work, the transcription of adenine riboswitch RNA was achieved by 3 step-, 6 step-, 8 step- and 10 step-PLOR reactions. The final products containing terminated (~100 nt) and full-length adenine riboswitch RNA (120 nt) at the last step of the PLOR reactions were loaded to 10–15% polyacrylamide analysis gels (Figure 2A). Because adenine riboswitch RNA used in this work is a transcription-regulatory RNA, the transcribed products are easily identified: a full-length RNA (marked as “FL” in Figure 2A) and a terminated RNA (marked as “T” in Figure 2A) lack of the terminator U-tract. The efficiency of termination in the 3 step-, 6 step-, 8 step- and 10 step-PLOR reactions was shown in Figure 2. The procedure and reagent usage in the four PLOR reactions were listed in Supplementary Tables S1–S4 and S8. Omitting at least one type of NTPs was applied to all except the last step in the PLOR reactions to pause the synthesis of the adenine riboswitch RNA as designed. Additionally, the pause positions at the 3 step-, 6 step-, 8 step and 10 step-PLOR are marked by blue, purple, red, and grey arrows, respectively (Figure 1A,B). The termination efficiency is defined as the fraction of T in the total transcripts (T+FL). As shown in Figure 2, the termination efficiency ranges from about 80% to 90% in the absence of adenine, higher than in the presence of adenine (about 50–60%) (Figure 2C). This matches the description of adenine riboswitch, in which the binding of adenine can trigger the transcription from termination to anti-termination (Figure 1). In our hands, the yields of the terminated RNA are higher than the full-length RNA in the absence or presence of 1 mM adenine (Figure 2B). The relative intensity of FL increased by about threefold during the 3-step PLOR upon the addition of 1 mM adenine (Figure 2B). The overall yields of PLOR can be calculated using the equation *E* = *E_i_* × *E_e_*^n^, where *E*, *E_i_* and *E_e_* represent the overall, initiation and elongation efficiency, respectively [24]. Additionally, n is the number of steps in PLOR except the initiation step, which is 2, 5, 7 and 9 in the 3-, 6-, 8- and 10-step PLOR reactions, respectively. According to the equation, the overall efficiency is inversely proportional to n, hence it is reasonable for us to observe that the overall transcription yields (the amounts of T and FL) decrease with the increase of PLOR steps, as seen in Figure 2B. However, the termination efficiency changes insignificantly among different PLOR reactions in the presence or absence of 1 mM adenine (Figure 2C). This indicates that the transcriptional termination of the adenine riboswitch insignificantly affects the pausing strategies of PLOR, although the total RNA yields decrease with the increase of PLOR steps. Among the four PLOR strategies, the termination efficiency and ratios of FL to T in the 8 step-PLOR affected mostly upon the addition of 1 mM adenine (Figure 2C,D). Without being noted, the 8 step-PLOR was used to test the effect of other factors on the transcription termination of adenine riboswitch.

### 2.2. The Effects of Adenine Concentration on Transcription Termination of Adenine Riboswitch RNA

**Figure 3:**
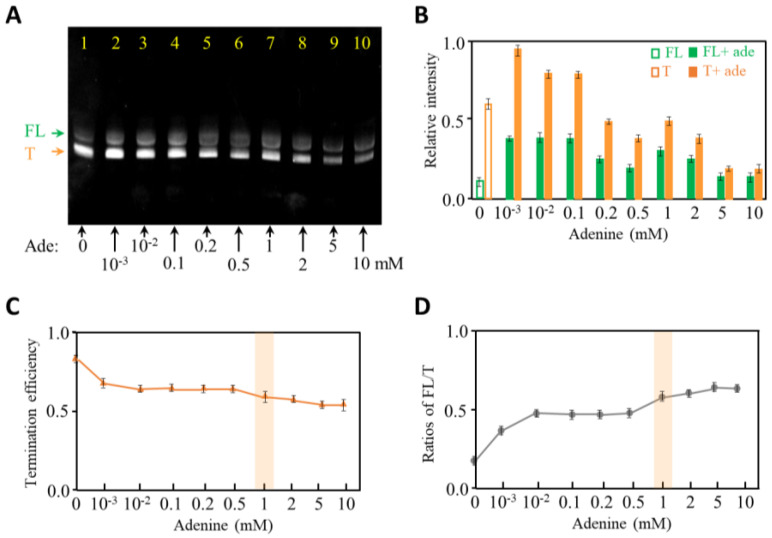
Transcription termination studies of adenine riboswitch at 0–10 mM adenine. (**A**) 12% denaturing PAGE gel image of the crude products generated from 8-step PLOR reactions in the presence of 0–10 mM adenine. The full-length and terminated products are marked by FL and T, respectively. The gel was irradiated under UV 260 nm. (**B**) The relative intensities of FL (in green) and T (in orange) produced at 0–10 mM adenine. The experiments were repeated three times. (**C**) Termination efficiency of adenine riboswitch transcription as a function of adenine concentration. (**D**) The ratios of FL to T of adenine riboswitch transcription as a function of adenine concentration.

Riboswitch can regulate gene expression by sensing its specific ligand among countless metabolites in cells [30,31]. As shown in Figure 1, the conformations of adenine riboswitch switch by the addition of adenine, which leads to the anti-termination of transcription. Our experimental results showed that the yields of terminated and full-length adenine riboswitch changed with adenine concentration (Figure 3 and Supplementary Table S5). More specifically, the termination efficiency decreases from about 85% to 60% as the adenine concentration increases from 0 to 10 mM (Figure 3C). Correspondingly, the ratios of FL to T increase from about 0.2 at 0 mM adenine to 0.6 at 10 mM adenine (Figure 3D). This matches Figure 1, in which the addition of adenine facilitates the anti-termination of adenine riboswitch RNA. In addition, the termination efficiency and ratios of FL/T change obviously from 0 to 0.01 mM adenine (Figure 3C,D). When we took a closer investigation on 0.05–10.0 μM adenine, we observed a negligible change in the termination efficiency at 0.05–1.0 μM adenine, following a continuous drop as adenine increased from 1.0 to 10.0 μM (Supplementary Figure S1). We anticipate that adenine riboswitch RNA tends to switch structures from terminator to anti-terminator at a low concentration of adenine, and 1 mM adenine is saturated for the structural change of the riboswitch. However, it is confusing that the production of adenine riboswitch decreases with the increase in adenine concentration. Without being noted, the comparison of transcription termination in the following experiments was performed with 1 mM adenine.

### 2.3. The Effects of Mg^2+^ Concentration on Transcription Termination of Adenine Riboswitch RNA

**Figure 4:**
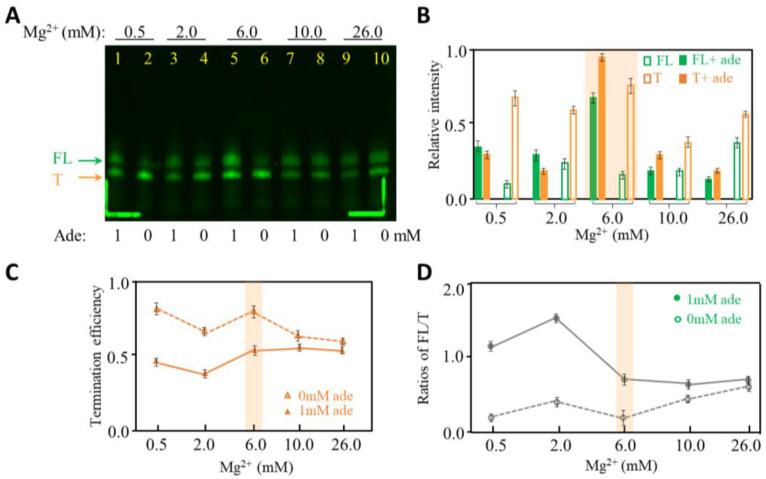
Transcription termination studies of adenine riboswitch at 0.5–26.0 mM Mg^2+^ in the presence of 0 or 1 mM adenine. (**A**) 12% denaturing PAGE gel image of the crude products generated at 0.5–26.0 mM Mg^2+^. The gel was irradiated under fluorescence 550 nm. (**B**) The relative intensities of FL (green) and T (orange) produced at 0.5–26.0 mM Mg^2+^ in the absence (in open columns) and presence of 1 mM adenine (in solid columns). The experiments were repeated three times. (**C**) Termination efficiency of adenine riboswitch transcription as a function of the Mg^2+^ concentration in the absence (in open triangles) and presence of 1 mM adenine (in solid triangles). (**D**) The ratios of FL to T of adenine riboswitch transcription as a function of the Mg^2+^ concentration in the absence (in open dots) and presence of 1 mM adenine (in solid dots).

Mg^2+^ is crucial to initiate in vitro transcription and RNA folding [32,33,34,35], and we then tested the effects of Mg^2+^ on the termination efficiency of the adenine riboswitch. In Figure 4, 0.5–26.0 mM Mg^2+^ were added to observe termination efficiency of the adenine riboswitch. To observe the transcribed RNA more sensitively, we used Cy3-labeled UTP instead of regular UTP at step four to introduce the fluorophore Cy3 to the adenine riboswitch by PLOR (Supplementary Table S6). By doing so, we can quantify the product amounts under fluorescence. Surprisingly, the overall yields of transcription do not increase continuously with Mg^2+^; instead, they reach maximum at 6.0 mM Mg^2+^ (Figure 4B). The termination efficiency is lower in the presence of 1 mM adenine than in the absence of adenine at 0.5–26.0 mM Mg^2+^ (Figure 4C). Additionally, the termination efficiency at 6.0 mM Mg^2+^ is about 85% in the absence of adenine, higher than at 10.0 or 26.0 mM Mg^2+^. The possible reason is that higher Mg^2+^ is speculated to speed up transcription and may be too fast for the adenine riboswitch to fold into the terminated conformation, hence reducing the termination efficiency. While in the presence of adenine, a high concentration of Mg^2+^ helps to stabilize the terminated and full-length conformation, which may offset the impact brought by speeding up transcription (Figure 4C,D). Additionally, based on our observation, 6.0 mM Mg^2+^ is conductive for terminating transcription and producing high amounts of both full-length and terminated products in the presence of 1 mM adenine. These results also suggest that the formation of terminator in adenine riboswitch RNA does not need Mg^2+^ to be too high.

### 2.4. The Effects of NTP Concentration on Transcription Termination of Adenine Riboswitch RNA

**Figure 5:**
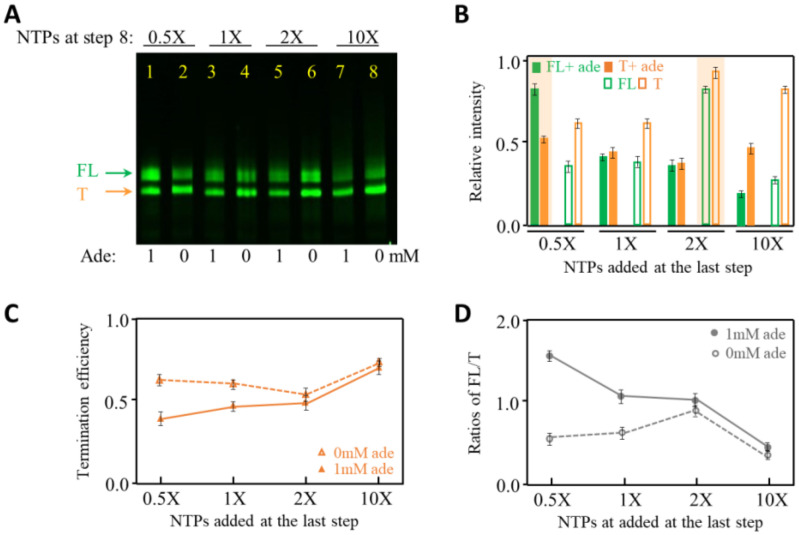
Transcription termination studies of adenine riboswitch at 0.5–10× NTPs in the last step of PLOR with 0 or 1 mM adenine. (**A**) 12% denaturing PAGE gel image of the crude products generated with 0.5–10× NTPs at the last step of PLOR in the presence of 0 or 1 mM adenine. (**B**) The relative intensities of FL (green) and T (orange) produced at 0.5–10× NTPs in the absence (in open columns) and presence of 1 mM adenine (in solid columns). The experiments were repeated three times. (**C**) The termination efficiency of adenine riboswitch transcription as a function of NTP concentration in the absence (in open triangles) and presence of 1 mM adenine (in solid triangles). (**D**) The ratios of FL to T of adenine riboswitch transcription as a function of NTP concentration in the absence (in open dots) and presence of 1 mM adenine (in solid dots).

To test NTP effects on transcription termination, we performed 8 step-PLOR reactions at different NTP concentrations in the last step. The NTP amounts are given in molar ratios, and 1× NTPs are the NTPs required exactly for producing one RNA molecule per DNA at an individual step of the PLOR. As shown in Figure 5A, the products with the addition of 0.5×, 1×, 2× and 10× NTPs at the last step in the absence and presence of 1 mM adenine were loaded. Please note that the NTP concentration added in step one was much higher than in other steps to efficiently initiate transcription in PLOR, and NTP usage is listed in Supplementary Table S7.

In the presence of adenine, less NTP at the last step facilitates to produce a higher amount of full-length RNA, and the NTP addition affects the yields of terminated RNA less significantly than the full-length RNA (Figure 5A,B). While in the absence of adenine, higher yields of both the full-length and terminated RNA products were observed at 2× NTPs (Figure 5A,B). As described earlier, the termination efficiency is lower in the presence of adenine than in the absence of adenine (Figure 5C), which is consistent with the ligand-sensitive nature of adenine riboswitch RNA. Interestingly, the highest termination efficiency of adenine riboswitch was obtained at 10× NTP in the absence and presence of 1 mM adenine (Figure 5C). Additionally, the addition of 1 mM adenine affects the termination efficiency and FL/T ratios mostly at 0.5× NTPs, and least at 10× NTPs (Figure 5D). This is probably because the transcription rate at 10× NTPs is so fast that the effect of adenine on structural folding of the adenine riboswitch is negligible. The results show that NTP has a great impact on the transcription yields and termination, and the effect of the ligand on the transcription termination of adenine riboswitch is more significant at low NTPs.

## 3. Discussion

Our results show that the single-pass transcription of PLOR reactions can be used to evaluate transcription termination after manually controlling the transcriptional process by omitting specific types of NTPs. Such a single-pass motif discontinues the transcription of nascent RNA, and the transcripts may reach folding equilibrium during the pause–restart period. Nonetheless, studying transcription by using PLOR has distinct advantages, for example, the immobilized DNA templates can be recycled economically and do not need to introduce radiolabeled labels. Instead, we can introduce fluorophores to improve detection sensitivity in PLOR without exposing to radioactive reagents, although fluorophores may be less sensitive than radioactive ^32^P in PAGE. Furthermore, it is important to note that our method has great flexibility in adjusting experimental conditions and tracking transcriptional outputs at subtle differences.

The simplicity and efficiency of the strategy make it a great alternative method for measuring transcription termination of various RNA systems and providing guidance for optimizing the yields of terminated and full-length RNA individually. Different from in vitro transcription, the RNA transcribed in our method discontinues, which can be potentially developed to study the interaction between RNAP, nascent RNA and the template, and also provides a co-transcriptional structural study of nascent RNA at different transcription stages. Furthermore, a more delicate design can be applied to our strategy, such as adding or removing ligands or cofactors only at one step to observe the instant effects of ligands or cofactors on a transcription system. Here, we only tested the adenine riboswitch system, but with the consideration that T7 RNAP has been widely used to synthesize countless RNA and PLOR has been used to synthesize multiple RNA, the strategy of applying PLOR to measure transcription efficiency should be suitable for various RNA.

## 4. Materials and Methods

### 4.1. Preparation of Solid-Phase DNA Templates for PLOR

DNA template with the sequence CCGCGGATGCGGAAAAAAAATCCTGATTACAAAAAATGTCATAAACAAATTTTGTAATCAGGATTTTACGGTTCCTGGTAGACACCCTCAAACCATATTATTGAGGTTATACAACTTCCCTATAGTGAGTCGTATTATGGACTAGCTGAATCAGA (Supplementary Table S1) was prepared by touchdown PCR (TD-PCR) reactions in the buffer (10 mM Tris-HCl, 50 mM KCl, 1.5 mM MgCl_2_, pH 8.0). The concentration of DNA templates, primers, dNTPs and Taq polymerase used in TD-PCR were 0.01 µM, 2 µM, 0.2 mM and 0.05 g/L. In our hands, TD-PCR has higher efficiency than regular PCR. TD-PCR contains the touchdown phase and the PCR phase. Twenty cycles are included in the touchdown phase to prevent nonspecific extension of primers, which begins with melting at 95 °C for 5 min, anneals at 75–45 °C for 45 s (the temperature drops 1.5 °C per cycle) and then elongates at 72 °C for 60 s. Thirty-five cycles are included in the PCR phase, which begins with melting at 95 °C for 30 s, anneals at 58 °C for 30 s and then elongates at 72 °C for 20 s. The sequences of the primers and templates used in the TD-PCR reactions are listed in Supplementary Table S1. It is worth pointing out that 5′-biotinylated forward primer was used to incorporate biotin into the DNA templates for subsequent immobilization on solid-phase beads. The PCR-generated DNAs were purified with 10% denaturing PAGE.

The biotin-DNA templates produced by PCR were then incubated with streptavidin-coated agarose beads (Smart-Life sciences, Changzhou, China) at 4 °C overnight in the buffer (40 mM Tris-HCl, 100 mM K_2_SO_4_, and 6 mM MgSO_4_, pH 8.0) to generate DNA-bead templates as reported elsewhere [23,24]. The solid-phase DNA-bead templates were stored at 4 °C before being used as templates in the PLOR reactions.

### 4.2. PLOR Reactions to Generate Terminated and Full-Length RNA

A total of four PLOR reactions, the 3 step-, 6 step-, 8 step- and 10 step-PLOR reactions, were performed. The detailed procedure and reagent usage were listed in Supplementary Tables S2–S8. Except step 1, the NTP amounts are based on the number of each type of NTP in an individual segment transcribed in a step. A much higher concentration of NTP was used at step 1 to initiate PLOR efficiently.

Here, we use the 8 step-PLOR as an example to illustrate the detailed PLOR procedure. The reagent usage is listed in Supplementary Tables S4–S7. T7 RNAP gently rotated with DNA-beads in the initiation buffer (40 mM Tris-HCl, 100 mM K_2_SO_4_, 6 mM MgSO_4_, 10 mM DTT, pH 8.0) at 37 °C for 10 min. The total reaction volume was 100 µL, and the concentration of T7 RNAP and DNA was 5 µM. At step 1 of PLOR, 400 µM ATP, 600 µM GTP and 64 µM UTP were added to the reaction and gently rotated with T7 RNAP and DNA-beads at 37 °C for 15 min, followed by filtration and rinsing the solid-phase reactants at least three times with the buffer (40 mM Tris-HCl, 6 mM MgSO_4_, pH 8.0). The residual steps were performed similarly to step 1 except different batches of NTPs were added and gently rotated with the solid-phase reactants in the buffer (40 mM Tris-HCl, 6 mM MgSO_4_, 0 or 1 mM adenine, pH 8.0) at 25 °C for 10 min. Additionally, the NTPs were added as follows: 25 μM ATP, 15 μM CTP and 20 μM UTP (step 2); 5 μM ATP, 35 μM GTP and 25 μM UTP (step 3); 10 μM ATP, 15 μM CTP and 5 μM UTP (step 4); 10 μM ATP, 10 μM CTP and 15 μM GTP (step 5); 20 μM ATP, 10 μM CTP and 15 μM UTP (step 6); 10 μM ATP, 5 μM GTP and 10 μM UTP (step 7). In order to test the effect of adenine, Mg^2+^ and NTP on transcription termination, the experimental conditions at step 8 were different: 60 μM ATP, 45 μM CTP, 45 μM GTP and 120 μM UTP in the buffer (40 mM Tris-HCl, 6 mM MgSO_4_, 0–10 mM adenine, pH 8.0) used for testing adenine (Supplementary Table S5); 60 μM ATP, 45 μM CTP, 45 μM GTP and 120 μM UTP in the buffer (40 mM Tris-HCl, 0.5~26 mM MgSO_4_, 0 or 1 mM adenine, pH 8.0) used for testing Mg^2+^ (Supplementary Table S6); 30–600 μM ATP, 22.5~450 μM CTP, 22.5–450 μM GTP and 60–1200 μM UTP in the buffer (40 mM Tris-HCl, 6 mM MgSO_4_, 0 or 1 mM adenine, pH 8.0) used for testing NTP concentration (Supplementary Table S7).

The 3 step-, 6 step- and 10 step-PLOR were performed similarly as the 8 step-PLOR reaction, except that different NTPs were added. Additionally, the NTP addition for 3 step-, 6 step- and 10 step-PLOR reactions was listed in Supplementary Tables S2, S3 and S8, respectively.

### 4.3. Quantification of Terminated and Full-Length RNA Products

The filtered RNA products at the final step were collected and loaded to 12% denaturing PAGE analysis gels for quantification analysis. The gels of unlabeled RNA were stained by Ultra Gel Red Nucleic Acid Stain (VAZYME Biotech, Nanjing, China) for 3–5 min, then irradiated at UV 260 nm in a Tanon 2500 gel imager (Tanon Science and Technology Co., Shanghai, China). The gels of Cy3-labeled RNA products were irradiated at fluorescence 550 nm without staining by the GenoSens fluorescent gel imager (Clinx Scientific Instrument Co., Shanghai, China). The gel bands were quantified using Image J software.

## Data Availability

The data presented in this study are available in the insert article or Supplementary Material provided here.

## Supplementary Materials

The following supporting information can be downloaded at: https://www.mdpi.com/article/10.3390/ijms24054934/s1.
