# Supplementary material for: Quantitative Analysis of Transcription Termination via Position-Selective Labeling of RNA (PLOR) Method
Source: Int J Mol Sci. 2023 Mar 3;24(5):4934. doi: 10.3390/ijms24054934 (PMC10003555; doi:10.3390/ijms24054934)
Supplement: Supplementary file 1 [file ijms-24-04934-s001.zip › ijms-2211100-supplementary.pdf]

Supplementary Information

Quantitative analysis of transcriptional termination by Position-selective Labeling of RNA (PLOR) method

Ping-yi Chien[1], Lingzhi Gao[1], and Yu Liu[1,*]

[1]State Key Laboratory of Microbial Metabolism, School of Life Science and Biotechnology, Shanghai Jiao Tong University, Shanghai, P. R. China.
[*]Corresponding author, E-mail: liuyu_sjtu@sjtu.edu.cn

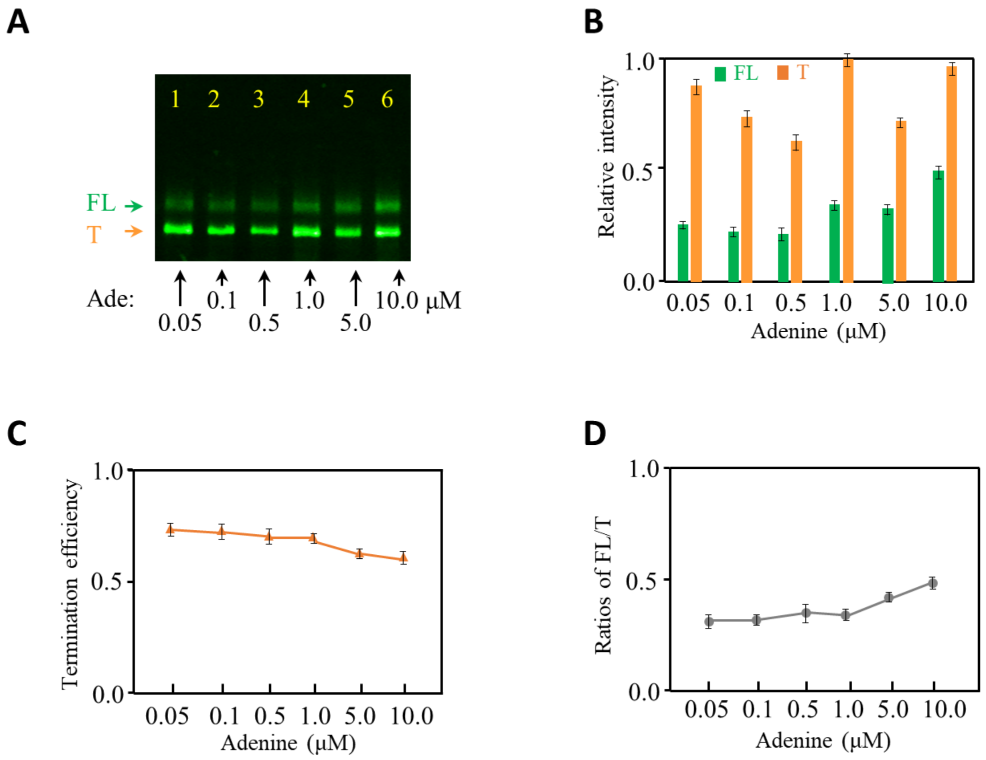

Supplementary Figure S1. Transcription termination studies of adenine riboswitch at 0.05~10.0 µM adenine. (A) Denaturing PAGE gel image of the crude products generated from 8-step PLOR reactions in the presence of 0.05~10.0 µM adenine. The full-length and terminated products are marked by FL and T respectively. The gel was irradiated under fluorescence 550 nm. (B) The relative intensities of FL (in green) and T (in orange) produced at 0.05~10.0 µM adenine. The experiments were repeated three times. (C) Termination efficiency of adenine riboswitch transcription as a function of adenine concentration. (D) The ratios of FL to T of adenine riboswitch transcription as a function of adenine concentration.

Supplementary Table S1: Sequences of DNA templates, primers and adenine riboswitch RNA

DNA/RNA sequences	Sequence 5'→3'
forward primer used in PCR	Biotin-TCTGATTCAGCTAGTCCATAATACGACT
reverse primer used in PCR	CCGCGGATGCGGAAAAAAAT
The non-coding strand of DNA template in PLOR	Biotin-*TCTGATTCAGCTAGTCCA*TAATACGACTCACTATA<br>GGGAAGTTGTATAACCTCAATAATATGGTTTGAGGGTG<br>TCTACCAGGAACCGTAAAATCCTGATTACAAAATTTGT<br>TTATGACATTTTTTGTAATCAGGATTTTTTTCCGCATCC<br>GCGG
The coding strand of DNA template in PLOR	CCGCGGATGCGGAAAAAAATCCTGATTACAAAAAAT<br>GTCATAAACAAATTTTGTAATCAGGATTTTACGGTTCCT<br>GGTAGACACCCTCAAACCATATTATTGAGGTTATACAA<br>CTTCCCTATAGTGAGTCGTATTA*TGGACTAGCTGAATCAG*<br>*A*
Adenine riboswitch RNA	GGGAAGUUGUAUAACCUCAAUAAUAUGGUUUGAGG<br>GUGUCUACCAGGAACCGUAAAAUCCUGAUUACAAAA<br>UUUGUUUAUGACAUUUUUUGUAAUCAGGAUUUUUU<br>UUCCGCAUCCGCGG

A linker (italicized) was added upstream of the T7 promoter (underlined).

Supplementary Table S2: Reagent usage in 5 µM, 100 µL 3-step PLOR

**Step 1: 37 °C, 15 min in the buffer (40 mM Tris-HCl, 100 mM K_2SO_4, 6 mM $MgSO_4$, 10 mM DTT, pH 8.0)**<br>Step 1: 400 µM ATP, 600 µM GTP, 64 µM UTP;<br><br>**Steps 2-3: 25 °C, 10 min in the buffer (40 mM Tris-HCl, 6 mM $MgSO_4$, 0 or 1 mM adenine, pH 8.0)**<br>Step 2: 25 µM ATP, 15 µM CTP, 20 µM UTP;<br>Step 3: 115 µM ATP, 80 µM CTP, 100 µM GTP, 175 µM UTP.

Supplementary Table S3: Reagent usage in 5 μM, 100 μL 6-step PLOR

Step 1: 37 °C, 15 min in the buffer (40 mM Tris-HCl, 100 mM K_2SO_4, 6 mM $MgSO_4$, 10 mM DTT, pH 8.0)
Step 1: 400 μM ATP, 600 μM GTP, 64 μM UTP;

Steps 2-6: 25 °C, 10 min in the buffer (40 mM Tris-HCl, 6 mM $MgSO_4$, 0 or 1 mM adenine, pH 8.0)
Step 2: 25 μM ATP, 15 μM CTP, 20 μM UTP;
Step 3: 5 μM ATP, 35 μM GTP, 25 μM UTP;
Step 4: 10 μM ATP, 15 μM CTP, 5 μM UTP;
Step 5: 10 μM ATP, 10 μM CTP, 15 μM GTP;
Step 6: 90 μM ATP, 55 μM CTP, 50 μM GTP, 145 μM UTP.

Supplementary Table S4: Reagent usage in 5 μM, 100 μL 8-step PLOR

Step 1: 37 °C, 15 min in the buffer (40 mM Tris-HCl, 100 mM K_2SO_4, 6 mM $MgSO_4$, 10 mM DTT, pH 8.0)
Step 1: 400 μM ATP, 600 μM GTP, 64 μM UTP;

Steps 2-8: 25 °C, 10 min in the buffer (40 mM Tris-HCl, 6 mM $MgSO_4$, 0 or 1 mM adenine, pH 8.0)
Step 2: 25 μM ATP, 15 μM CTP, 20 μM UTP;
Step 3: 5 μM ATP, 35 μM GTP, 25 μM UTP;
Step 4: 10 μM ATP, 15 μM CTP, 5 μM UTP;
Step 5: 10 μM ATP, 10 μM CTP, 15 μM GTP;
Step 6: 20 μM ATP, 10 μM CTP, 15 μM UTP;
Step 7: 10 μM ATP, 5 μM GTP, 10 μM UTP;
Step 8: 60 μM ATP, 45 μM CTP, 45 μM GTP, 120 μM UTP.

Supplementary Table S5: Reagent usage in 5 μM, 100 μL 8-step PLOR for testing adenine

Step 1: 37 °C, 15 min in the buffer (40 mM Tris-HCl, 100 mM K_2SO_4, 6 mM $MgSO_4$, 10 mM DTT, pH 8.0)
Step 1: 400 μM ATP, 600 μM GTP, 64 μM UTP;

Steps 2-7: 25 °C, 10 min in the buffer (40 mM Tris-HCl, 6 mM $MgSO_4$, 0~10 mM adenine, pH 8.0)
Step 2: 25 μM ATP, 15 μM CTP, 20 μM UTP;
Step 3: 5 μM ATP, 35 μM GTP, 25 μM UTP;
Step 4: 10 μM ATP, 15 μM CTP, 5 μM UTP;
Step 5: 10 μM ATP, 10 μM CTP, 15 μM GTP;
Step 6: 20 μM ATP, 10 μM CTP, 15 μM UTP;
Step 7: 10 μM ATP, 5 μM GTP, 10 μM UTP;

Steps 8: 25 °C, 10 min in the buffer (40 mM Tris-HCl, 6 mM $MgSO_4$, 0~10 mM adenine, pH 8.0)
Step 8: 60 μM ATP, 45 μM CTP, 45 μM GTP, 120 μM UTP.

Supplementary Table S6: Reagent usage in 5 μM, 100 μL 8-step PLOR for testing Mg^{2+}

Step 1: 37 °C, 15 min in the buffer (40 mM Tris-HCl, 100 mM K_2SO_4, 6 mM $MgSO_4$, 10 mM DTT, pH 8.0)
Step 1: 400 μM ATP, 600 μM GTP, 64 μM UTP;

Steps 2-7: 25 °C, 10 min in the buffer (40 mM Tris-HCl, 6 mM $MgSO_4$, 0 or 1 mM adenine, pH 8.0)
Step 2: 25 μM ATP, 15 μM CTP, 20 μM UTP;
Step 3: 5 μM ATP, 35 μM GTP, 25 μM UTP;
Step 4: 10 μM ATP, 15 μM CTP, 5 μM Cy3-UTP (30 °C, 10 min);
Step 5: 10 μM ATP, 10 μM CTP, 15 μM GTP;
Step 6: 20 μM ATP, 10 μM CTP, 15 μM UTP;
Step 7: 10 μM ATP, 5 μM GTP, 10 μM UTP;

Steps 8: 25 °C, 10 min in the buffer (40 mM Tris-HCl, 0.5~26 mM $MgSO_4$, 0 or 1 mM adenine, pH 8.0)
Step 8: 60 μM ATP, 45 μM CTP, 45 μM GTP, 120 μM UTP.

Supplementary Table S7: Reagent usage in 5 μM, 100 μL 8-step PLOR for testing NTPs

**Step 1: 37 °C, 15 min in the buffer (40 mM Tris-HCl, 100 mM K_2SO_4, 6 mM $MgSO_4$, 10 mM DTT, pH 8.0)**
Step 1: 400 μM ATP, 600 μM GTP, 64 μM UTP;
**Steps 2-7: 25 °C, 10 min in the buffer (40 mM Tris-HCl, 6 mM $MgSO_4$, 0 or 1 mM adenine, pH 8.0)**
Step 2: 25 μM ATP, 15 μM CTP, 20 μM UTP;
Step 3: 5 μM ATP, 35 μM GTP, 25 μM UTP;
Step 4: 8 μM ATP, 12 μM CTP, 4 μM Cy3-UTP (30 °C, 10 min);
Step 5: 10 μM ATP, 10 μM CTP, 15 μM GTP;
Step 6: 20 μM ATP, 10 μM CTP, 15 μM UTP;
Step 7: 10 μM ATP, 5 μM GTP, 10 μM UTP;
**Steps 8: 25 °C, 10 min in the buffer (40 mM Tris-HCl, 6 mM $MgSO_4$, 0 or 1 mM adenine, pH 8.0)**
Step 8 for 0.5X NTP: 30 μM ATP, 22.5 μM CTP, 22.5 μM GTP, 60 μM UTP;
Step 8 for 1X NTP: 60 μM ATP, 45 μM CTP, 45 μM GTP, 120 μM UTP;
Step 8 for 2X NTP: 120 μM ATP, 90 μM CTP, 90 μM GTP, 240 μM UTP;
Step 8 for 10X NTP: 600 μM ATP, 450 μM CTP, 450 μM GTP, 1.2 mM UTP.

Supplementary Table S8: Reagent usage in 5 μM, 100 μL 10-step PLOR

**Step 1: 37 °C, 15 min in the buffer (40 mM Tris-HCl, 100 mM K_2SO_4, 6 mM $MgSO_4$, 10 mM DTT, pH 8.0)**
Step 1: 400 μM ATP, 600 μM GTP, 64 μM UTP;
**Steps 2-10: 25 °C, 10 min in the buffer (40 mM Tris-HCl, 6 mM $MgSO_4$, 0 or 1 mM adenine, pH 8.0)**
Step 2: 25 μM ATP, 15 μM CTP, 20 μM UTP;
Step 3: 5 μM ATP, 35 μM GTP, 25 μM UTP;
Step 4: 10 μM ATP, 15 μM CTP, 5 μM UTP;
Step 5: 10 μM ATP, 10 μM CTP, 15 μM GTP;
Step 6: 20 μM ATP, 10 μM CTP, 15 μM UTP;
Step 7: 10 μM ATP, 5 μM GTP, 10 μM UTP;
Step 8: 20 μM ATP, 5 μM CTP, 15 μM UTP;
Step 9: 10 μM ATP, 10 μM GTP, 20 μM UTP;
Step 10: 30 μM ATP, 40 μM CTP, 35 μM GTP, 85 μM UTP.
